# Good Manufacturing Practice-Compliant Production and Lot-Release of *Ex Vivo* Expanded Regulatory T Cells As Basis for Treatment of Patients with Autoimmune and Inflammatory Disorders

**DOI:** 10.3389/fimmu.2017.01371

**Published:** 2017-10-26

**Authors:** Manuel Wiesinger, Diane Stoica, Susanne Roessner, Carmen Lorenz, Anika Fischer, Raja Atreya, Clemens F. Neufert, Imke Atreya, Alexander Scheffold, Beatrice Schuler-Thurner, Markus F. Neurath, Gerold Schuler, Caroline J. Voskens

**Affiliations:** ^1^Department of Dermatology, Friedrich-Alexander Universität Erlangen-Nürnberg, Erlangen, Germany; ^2^Department of Medicine 1, Friedrich-Alexander Universität Erlangen-Nürnberg, Erlangen, Germany; ^3^Department of Cellular Immunology, Clinic for Rheumatology and Clinical Immunology, Charité—University Medicine Berlin, Berlin, Germany

**Keywords:** regulatory T cell, good manufacturing practice, autoimmunity, expansion, lot-release

## Abstract

In recent years, the exploration of regulatory T cell (Treg)-based cellular therapy has become an attractive strategy to ameliorate inflammation and autoimmunity in various clinical settings. The main obstacle to the clinical application of Treg in human is their low number circulating in peripheral blood. Therefore, *ex vivo* expansion is inevitable. Moreover, isolation of Treg bears the risk of concurrent isolation of unwanted effector cells, which may trigger or deteriorate inflammation upon adoptive Treg transfer. Here, we present a protocol for the GMP-compliant production, lot-release and validation of *ex vivo* expanded Tregs for treatment of patients with autoimmune and inflammatory disorders. In the presented production protocol, large numbers of Treg, previously enriched from a leukapheresis product by using the CliniMACS^®^ system, are *ex vivo* expanded in the presence of anti-CD3/anti-CD28 expander beads, exogenous IL-2 and rapamycin during 21 days. The expanded Treg drug product passed predefined lot-release criteria. These criteria include (i) sterility testing, (ii) assessment of Treg phenotype, (iii) assessment of non-Treg cellular impurities, (iv) confirmation of successful anti-CD3/anti-CD28 expander bead removal after expansion, and (v) confirmation of the biological function of the Treg product. Furthermore, the Treg drug product was shown to retain its stability and suppressive function for at least 1 year after freezing and thawing. Also, dilution of the Treg drug product in 0.9% physiological saline did not affect Treg phenotype and Treg function for up to 90 min. These data indicate that these cells are ready to use in a clinical setting in which a cell infusion time of up to 90 min can be expected. The presented production process has recently undergone on site GMP-conform evaluation and received GMP certification from the Bavarian authorities in Germany. This protocol can now be used for Treg-based therapy of various inflammatory and autoimmune disorders.

## Introduction

Regulatory T cells (Treg) play a critical role in maintaining immune homeostasis and limiting autoimmune responses by modulation of both innate and adaptive immunity (1). Classically defined Treg are characterized by their constitutive expression of CD4, CD25, and FoxP3 (2) and nearly absent expression of CD127 (3, 4). They can be divided in (i) natural Treg originating from the thymus and peripherally induced Treg, which differentiate from naïve T cells when self or non-self antigen is encountered under tolerogenic conditions (5, 6). Their existence in humans has first been described in 2001, when several groups were able to isolate (7–9) and expand (10) suppressive CD4^+^CD25^+^ T cells from human peripheral blood. Animal studies have shown that Treg successfully prevent type I diabetes, experimental autoimmune encephalitis, rheumatoid arthritis, inflammatory bowel disease, systemic lupus erythematosus, scurfy disease, graft-versus-host disease, and transplant rejection (11). As a result, the exploration of Treg-based cellular therapy has become an attractive strategy to induce tolerance in various clinical settings in patients (12). However, the main obstacle to clinical application of Treg in humans is their low number circulating in peripheral blood. Therefore, initial Treg enrichment and subsequent expansion protocols are necessary to generate clinical relevant Treg numbers. Treg enrichment from a peripheral blood product is challenging, since activated conventional human T cells may also express CD25 (13). As a result, isolation of Treg bears the risk of concurrent isolation of unwanted effector cells.

Currently, three main strategies to isolate and expand highly enriched Treg populations from a human blood product are exploited by several research groups. First, Treg can be isolated and expanded from a donor-derived umbilical cord blood product (14–17), yet this approach is not feasible in other settings than stem cell transplantation, since it cannot be excluded that allogeneic donor-derived Treg itself induce graft-versus-host like reactions in non-transplant patients. Alternatively, highly enriched Treg populations can be isolated using good manufacturing practice (GMP)-approved flow cytometry-based (FACS) cell sorters (18–20) or the mTOR inhibitor rapamycin can be added to the cell culture process to inhibit the proliferation of contaminating effector T cells (21–23) The latter is a calcineurin inhibitor that is widely used to prevent allograft rejection after transplantation (24). Previous animal studies showed that rapamycin decreases the number of CD4^+^ cell subsets in mice, but increases the number of functional Treg (25). Based on these findings, Ogino et al. provided the proof-of-concept that mouse CD4^+^ T cells can be expanded *ex vivo* in the presence of rapamycin (26). The addition of rapamycin to the cell cultures affected overall expansion efficiency but effectively inhibited the outgrowth of non-suppressive effector T cells. In addition, the rapamycin-expanded Treg ameliorated colitis in an SCID mouse model.

Safinia et al. (27) were the first to establish a GMP-compliant production protocol to expand CD25^+^-enriched cells from peripheral blood in the presence of rapamycin with the intention to prevent rejection after liver transplantation. In their 36-day expansion protocol, multiple rounds of *in vitro* Treg stimulation are necessary to reach clinically relevant Treg numbers. This may result in loss of FoxP3 expression and epigenetic stability, thus increasing the risk of *in vivo* Treg conversion into unwanted inflammatory effector cells.

Here, we provide the CD25^+^ enrichment protocol, *ex vivo* expansion protocol as well as the validated lot-release protocols that have been approved by the German regulatory authorities for a Treg drug product intended for clinical use in patients with autoimmune and inflammatory disorders. Treg produced by this 21-day protocol are epigenetically stable, suppressive and contain less than 0.1% of contaminating CD8^+^ effector cells. Moreover, we demonstrate the stability of the Treg drug product both after storage for up to 12 months and after subsequent dilution in a 0.9% physiological saline infusion solution. Also, we show that the Treg drug product remains polyclonal after 21 days of expansion and expresses various receptors associated with lymphocyte trafficking to secondary lymphoid organs and sites of inflammation. The protocol is scheduled to produce Treg for a phase I dose-escalation in patients and serves as an add-on platform for the adoptive transfer of Treg in a broad range of autoimmune and inflammatory disorders.

## Material and Methods

### Ethical Considerations

This study was approved by the local Institutional Review Board (IRB) of the Friedrich-Alexander-Universität Erlangen-Nürnberg under IRB number 151_12 B. In agreement with IRB approval and in accordance with the Declaration of Helsinki, oral and written consent was obtained from all healthy donors who donated blood for this study.

### Materials and Equipment

The following materials are used during the Treg production process:

**Table:** 

Autologous leucapherisate	
Autologous plasma	
MACS^®^ GMP ExpAct Treg Kit	Miltenyi Biotec (# 170-076-119)
Human serum albumin	Baxter (# PL 00116/0620)
MACS^®^ GMP Rapamycin	Miltenyi Biotec (# 170-076-308)
CliniMACS^®^ CD8 Reagent	Miltenyi Biotec (# 275-01)
CliniMACS^®^ CD19 Reagent	Miltenyi Biotec (# 179-01)
CliniMACS^®^ CD25 Reagent	Miltenyi Biotec (# 274-01)
l-Glutamine	Lonza (# BE 17-605 E)
CliniMACS^®^ PBS/EDTA	Miltenyi Biotec (# 700-25)
IL-2 (Proleukin^®^)	Novartis Pharma (# PZN 02238131)
X-VIVO15	Lonza (# BE 04-744)
Dimethyl sulfoxide (DMSO)	Sigma-Aldrich (# D2438)
Glucose solution 40% (Glucosteril 40%)	Frescenius Kabi Deutschland GmbH

### Treg Manufacture

**Figure 1:**
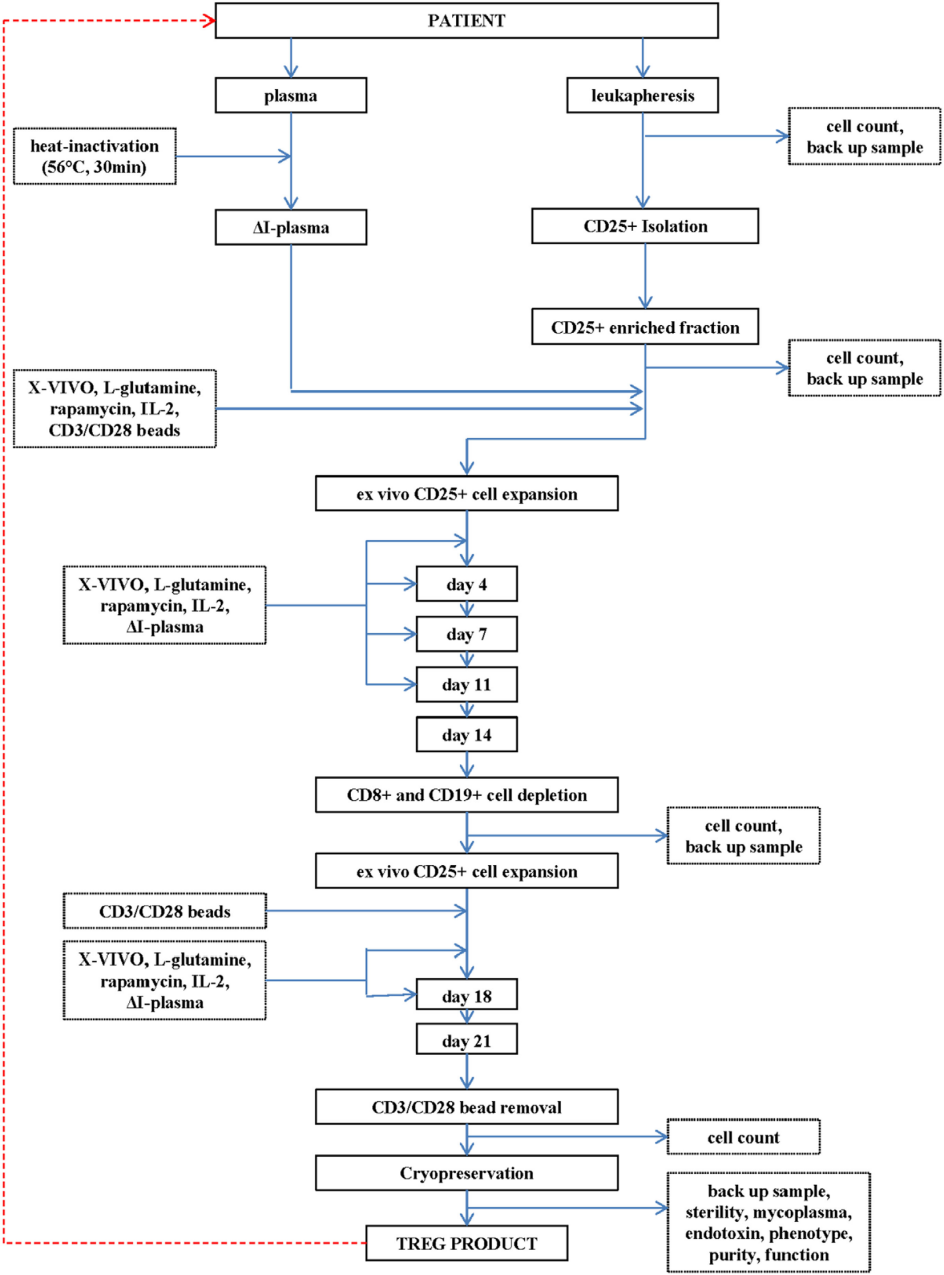
Flowchart of the production of the regulatory T cell (Treg) drug substance and Treg drug product.

A detailed overview of the manufacturing process is provided in Figure 1. The complete manufacturing process is performed in the GMP facility of the department of dermatology at the Friedrich-Alexander Universität Erlangen-Nürnberg. The manufacturing process is approved by the Bavarian Authorities under number DE_BY_05_MIA_2017_0012/55.2-2678.3-41-4-16. All cell purification steps are performed by using a CliniMACS^®^ system (Miltenyi Biotec, Bergisch Gladbach, Germany) in conjunction with ISO certified CliniMACS^®^ CD8 (Miltenyi Biotec, 275-01), CD19 (Miltenyi Biotec, 179-01), and CD25 (Miltenyi Biotec, 274-01) bead reagents. All purification steps are performed with GMP-grade CliniMACS^®^ PBS/EDTA buffer (Miltenyi Biotec, 700-25) supplemented with clinical grade human serum albumin (Baxter, PL 00116/0620, PEI.H.03272.01-1). This buffer is hereafter called PBS–HSA–EDTA. All cell culture steps were performed in the presence of X-VIVO 15 medium without gentamicin and phenol red (Lonza, BE 04-744) supplemented with heat inactivated autologous plasma, clinical grade IL-2 (1,000 IU/ml, Proleukin^®^ S, Aldesleukin, Novartis Pharma, PZN 02238131), MACS^®^ GMP rapamycin (100 ng/ml, Miltenyi Biotec, 170-076-308), and l-glutamine (200 mM, Lonza, BE 17-605 E). This medium is hereafter called complete autologous culture medium.

A leukapheresis product (department of Transfusion Medicine, Friedrich-Alexander Universität Erlangen-Nürnberg, Erlangen, Germany) is used as cell source for initial CD25^+^ cell enrichment.

#### CD25^+^ Cell Isolation

The operating procedures to enrich or deplete select cell subsets from a leukapheresis product are standardized and provided by the manufacturer of the CliniMACS^®^ device (Miltenyi Biotec). Upon arrival in the GMP facility, the leucapherisate is diluted 1 + 3 with PBS–HSA–EDTA buffer and subsequently centrifuged at 200 *g* for 15 min at 22°C. After centrifugation, the supernatant is removed, the leucapherisate is resuspended in PBS–HSA–EDTA and centrifuged at 300 *g* for 15 min at 4°C. After this centrifugation step and subsequent removal of the supernatant, the leucapherisate is resuspended in 380 ml of cold PBS–HSA–EDTA and labeled with CliniMACS^®^ CD25 reagent. The CliniMACS^®^ CD25 bead reagent specifically labels up to 600 × 10^6^ CD25^+^ cells within a total population consisting of maximal 40 × 10^9^ white blood cells. These CliniMACS^®^ acceptance criteria are provided by Miltenyi Biotec. If one of these acceptance criteria is not met, a maximum of two portions, instead of one portion, of CliniMACS CD25 bead reagent are used to specifically label the leucapherisate. CD25-labeling is performed during 15 ± 2 min at 2–8°C at a cell shaker programmed at 25 rpm. After labeling is completed, the cell suspension is washed with PBS–HAS–EDTA, diluted in 100 ml PBS–HSA–EDTA and transferred into a cell collection bag. CD25^+^ enrichment is performed by using the automatic CliniMACS^®^ ENRICHMENT 3.2 program of the CliniMACS^®^ device according to the manufacturer’s instructions. The CD25^+^ enriched cell fraction is used for further manufacturing.

### Start of CD25^+^ Cell Expansion on Day 0

Cell count and viability of the CD25^+^ cells was determined on two samples of 40 µl by trypan blue staining according to European Pharmacopeia (Ph. Eur.) 2.7.29. Depending on cell number, CD25^+^ cells were seeded at a density of 0.5 × 10^5^ cells/ml in a 24-well or 6-well culture plate in complete autologous culture medium. To facilitate *in vitro* CD25^+^ cell expansion, clinical grade IL-2 (1,000 IU/ml), rapamycin (100 ng/ml), and anti-CD3/anti-CD28 expander beads (MACS^®^ GMP ExpAct Treg Kit, Miltenyi Biotec) were added at a bead-to-cell ratio of 4:1 to the cell cultures. Cell cultures were gently mixed and incubated at 37 ± 1°C, 5 ± 1% CO_2_, >70% r.h. for 4 days.

#### Addition of Supplements at Day 4

At day 4, fresh IL-2 (1,000 IU/ml) and rapamycin (100 ng/ml) were added to the cell cultures to substitute for cellular consumption. Cell cultures were gently mixed and incubated at 37 ± 1°C, 5 ± 1% CO_2_, >70% r.h. for 3 days.

#### Addition of Supplements and Medium on Day 7

At day 7, cell culture plates were collected from the incubator, and the total cell culture volume transferred into a T75 flask. Cell count and viability of the CD25^+^ cells was determined on two samples of 40 µl by trypan blue staining according to Ph. Eur. 2.7.29. Depending on cell number, the cell density was adjusted to 0.5 × 10^6^ cells/ml by adding fresh complete autologous culture medium, and the cell suspension was seeded in new culture plates. To substitute for cellular consumption, fresh IL-2 (1,000 IU/ml) and rapamycin (100 ng/ml) were added. Cell cultures were gently mixed and incubated at 37 ± 1°C, 5 ± 1% CO_2_, >70% r.h. for 4 days.

#### Addition of Supplements and Medium on Day 11

Analogous to day 7, cell culture plates were collected from the incubator, and the cell suspension, depending on the volume, transferred into a T75 or 1 l cell culture flask. Cell count and viability of the CD25^+^ cells was determined on two samples of 40 µl by trypan blue staining according to Ph. Eur. 2.7.29. Depending on cell number, the cell density was adjusted to 0.5 × 10^6^ cells/ml by adding fresh complete autologous culture medium. Depending on total volume, the cell suspension was seeded in T75 or T175 cell culture flasks. To substitute for cellular consumption, fresh IL-2 (1,000 IU/ml) and rapamycin (100 ng/ml) were added. Cell cultures were gently mixed and incubated at 37 ± 1°C, 5 ± 1% CO_2_, >70% r.h. for 3 days.

#### Depletion of CD8^+^ and CD19^+^ Cells on Day 14

At day 14, cell culture flasks were collected from the incubator, and the cell suspension, depending on the volume, transferred into a T75 or 1 l cell culture flasks. Cell count and viability of the CD25^+^ cells was determined on two samples of 40 µl by trypan blue staining according to Ph. Eur. 2.7.29. To deplete potentially contaminating CD8^+^ and CD19^+^ cells, the CD25^+^ cell product was magnetically labeled with CliniMACS^®^ CD8 and CliniMACS^®^ CD19 bead reagent according to the manufactures’ instructions. Specifically, up to 4 × 10^9^ CD8^+^ cells and up to 5 × 10^9^ CD19^+^ cells within a total population consisting of maximal 40 × 10^9^ cells may specifically be labeled by 7.5 ml of the CliniMACS^®^ CD8 bead reagent and CliniMACS^®^ CD19 bead reagent, respectively. These CliniMACS^®^ acceptance criteria were provided by Miltenyi Biotec. In general, CD25^+^ cell expansion on day 14 does not result in total cell numbers above 1 × 10^9^ total cells, and the relative contamination with CD8^+^ and/or CD19^+^ cells is assumed to be below 25%. Therefore, a predefined aliquot of 1.875 ml of CliniMACS^®^ CD8 bead reagent and CliniMACS^®^ CD19 bead reagent is used to label up to 1.0 × 10^9^ CD8^+^ cells and up to 1.25 × 10^9^ CD19^+^ cells within a total population consisting of maximal 10 × 10^9^ cells. For depletion of CD8^+^ and CD19^+^ cells, sterile CliniMACS^®^ PBS/EDTA buffer supplemented with human serum albumin is used. Depletion of CD8^+^ and CD19^+^ cells is performed by using the automatic CliniMACS^®^ DEPLETION 2.1 program of the CliniMACS^®^ device. This program facilitates automated magnetic depletion of CD8^+^ and CD19^+^ cells in a closed, sterile system. Two cell fractions are collected into bags according to the instrumental settings of the CliniMACS^®^ DEPLETION 2.1 program. The CD8^+^- and CD19^+^-depleted cell fraction 2 is used for further processing.

Cell number in fraction 2 is determined on two samples of 40 µl by trypan blue staining according to Ph. Eur. 2.7.29. CD25^+^/CD8^−^/CD19^−^ cells of fraction 2 obtained from the CliniMACS^®^ device are continued to be cultivated *in vitro*. Depending on cell number, the cell density was adjusted to 0.5 × 10^6^ cells/ml by adding fresh complete autologous culture medium. Depending on total volume, the cell suspension was seeded in T75 or T175 cell culture flasks. To compensate for any anti-CD3/anti-CD28 bead removal during the CD8^+^ and CD19^+^ cell depletion process, anti-CD3/anti-CD28 beads (analogously to the amount of beads used at day 0) are added to the cell cultures. Finally, fresh IL-2 (1,000 IU/ml) and rapamycin (100 ng/ml) were added. Cell cultures were gently mixed and incubated at 37 ± 1°C, 5 ± 1% CO_2_, >70% r.h. for 4 days.

#### Addition of Supplements and Medium on Day 18

Analog to days 7 and 11, cell culture plates or flasks were collected from the incubator, and the cell suspension, depending on the volume, transferred into a T75 or 1 l cell culture flasks. Cell count and viability of the CD25^+^ cells was determined on two samples of 40 µl by trypan blue staining according to Ph. Eur. 2.7.29. Depending on cell number, the cell density was adjusted to 0.5 × 10^6^ cells/ml by adding fresh complete autologous culture medium. Depending on total volume, the cells are subsequently seeded in T75 or T175 cell culture flasks. To substitute for cellular consumption, fresh IL-2 (1,000 IU/ml) and rapamycin (100 ng/ml) are added. Cell cultures are gently mixed and incubated (37 ± 1°C, 5 ± 1% CO_2_, >70% r.h.) for an additional 3 days.

#### Harvesting of CD25^+^ Cells at Day 21

At day 21, cell culture plates or flasks are collected from the incubator, and the total cell culture volume is transferred into a set of 50 ml centrifuge tubes. Culture flasks are washed once with approximately 10–20 ml of PBS/EDTA buffer supplemented with human serum albumin. Used washing buffer is also transferred into the 50 ml centrifuge tubes. Tubes are centrifuged, supernatants are discarded, and pellets are collected into a set of 50 ml centrifuge tubes by resuspending pellets with approximately 5 ml of PBS/EDTA buffer supplemented with human serum albumin. Cell count and cell viability of CD25^+^/CD8^−^CD19^−^ cells are determined on two samples of 40 µl by trypan blue staining according to Ph. Eur. 2.7.29.

#### Anti-CD3/Anti-CD28 Expander Bead and CD25-, CD8-, and CD19-Labeling Bead Removal on Day 21

A maximum of 2.04 × 10^10^ beads can be depleted from a maximum of 4 × 10^10^ CD25^+^ cells in a final concentration of 20 × 10^6^–400 × 10^6^/ml during the bead removal process. These CliniMACS^®^ acceptance criteria are provided by Miltenyi Biotec. In general, CD25^+^ cell expansion on day 21 does not result in total cell numbers above 4 × 10^10^ total cells. Therefore, the assumed maximum amount of added beads is 0.48 × 10^10^ [four times the maximum amount of isolated CD25^+^ cells at day 0 (1,200 × 10^6^)]. For bead removal, sterile CliniMACS PBS/EDTA buffer supplemented with human serum albumin is used. Bead removal is performed by using the automatic CliniMACS^®^ DEPLETION 2.1 program of the CliniMACS^®^ device. The CliniMACS^®^ device facilitates automated magnetic depletion of anti-CD3/anti-CD28 expander beads and CD25-, CD8-, and CD19-labeling beads in a closed, sterile system. Two cell fractions are collected into bags according to the instrumental settings of the CliniMACS^®^ DEPLETION 2.1 program. The bead-depleted cell fraction 2 is used for filling and storage.

#### Filling and Storage of the CD25^+^ Cells at Day 21

The bead-depleted cells are transferred into a set of 50 ml centrifuge tubes. Tubes are centrifuged, supernatants are discarded, and pellets are collected into 100 ml of PBS/EDTA buffer supplemented with human serum albumin. The final cell number in cell fraction 2 after bead removal is determined on two samples of 40 µl by trypan blue staining according to Ph. Eur. 2.7.29. Before filling and storage, a freezing medium is freshly prepared in a sterile bottle. The total volume is calculated based on the cell counting results obtained after the first centrifugation step after the anti-CD3/anti-CD28 bead removal step. The freezing medium consists of human serum albumin, DMSO, and 40% glucose solution (hereafter called freezing medium). After definition of the final CD25^+^ cell number, cells are centrifuged and depending on cell number resuspended in an appropriate volume of human serum albumin at a final concentration of 20 × 10^6^ viable cells/ml. Five hundred microliters of cell suspension are filled into each cryovial (1.0 ml total volume). After addition of 500 µl freezing medium (55.5 Vol.-% human serum albumin, 25.0 Vol.-% DMSO, and 20.0 Vol.-% glucose) to every vial, the closed vials are mixed gently and transferred immediately into a freezing container. The container is than stored immediately at −75 ± 10°C for 4–18 h. The vials are transferred to the gas phase of liquid nitrogen (≤−150°C) for up to 2 years.

### Treg Lot-Release

#### Assessment of Treg Drug Product Identity and Cellular Composition

Throughout the manuscript, Treg are defined as CD4^+^CD25^+^CD127^−^ cells (=Treg drug product identity) based on previously published data (3, 4). Treg drug product identity was determined before and after 21 days of expansion by staining with directly conjugated mouse antihuman antibodies (mAbs) against CD4 (FITC, clone RPA-T4), CD25 (FITC, clone M-A251), and CD127 (PE, clone RDR5). Treg drug product cellular composition was determined before and after 21 days of expansion by staining with directly conjugated mouse antihuman mAbs against CD8 (FITC, clone SK1), CD19 (FITC, clone SJ25C1), CD16 (PE, clone 3G8), and CD56 (PE, clone B159). Corresponding IgG_1,κ_ mouse isotype controls (FITC, clone MOPC-21 and PE, clone MOPC-21) were included to assess unspecific binding. Potential dead cells were excluded by labeling with propidium iodide (PI) (BD Biosciences) according to the manufacturer’s instructions. Cells were acquired using a FACS Calibur (BD Biosciences), and data were analyzed using CellQuest™ Pro (BD Biosciences) software. For information-only purposes, cells were intracellularly stained with FoxP3 (clone PCH101) using a FoxP3/Transcription Staining Buffer Set (eBioscience, San Diego, CA, USA) according to the manufacturer’s instructions.

In general, flow cytometry is performed as described in Ph. Eur. 2.7.24. The specific method for flow cytometry was validated, since it is not in detail described in the Ph. Eur. In brief, thawed dendritic cells were used to validate the method for flow cytometry. Thawed dendritic cells, thawed lymphocytes, thawed Treg, and thawed CD25^−^ cells were used to determine specificity. Lymphocytes were single stained with CD4, CD127, or CD8 antibody, respectively, fixed with 1% formaldehyde, washed twice and subsequently mixed with unstained lymphocytes of the same preparation. To determine specificity of the CD25 antibody, Treg were single stained with the CD25 antibody, fixed with 1% formaldehyde, washed twice and subsequently mixed with unstained Treg of the same preparation The FACS result for stained lymphocytes or Treg was set to 1 (=100% positive). For specificity purposes, optimal positive and negative cells for each antibody characterizing the Treg drug product were measured. Nominal negative cells shall show less than 0.04 (=4%) positive cells, and nominal positive cells shall show more than 0.04 (=4%) positive ones. Summarized results are shown in Table 1. In addition, the stringent cutoff of less than 0.1% contaminating CD8^+^ cells in the final Treg product was validated separately. To avoid false negative CD8^+^ values in the final Treg product, the number of to acquire cells was increased to 300,000. A total of six acquisitions were performed with mature monocyte-derived dendritic cells, which represented the CD8^−^ cell subset. The SD (*s*) was calculated from all acquired “CD8^+^” cells within the CD8^−^ mature dendritic cell subset, and the quantification limit (QL) was calculated based on the following formula: QL = 10*s*/*a* with *a* set to 0.973 (=linear regression value derived from the flow cytometry validation report) (data not shown).

**Table 1:** Validation results of analysis by flow cytometry.

Validation part	Parameter	Acceptance	Result		Passed
Reproducibility	Coefficient of variation	≤5%	0.3	2.0%	X yes □ no
			0.5	2.5%	
			0.7	0.8%	

Intermediate precision	Deviation of mean	≤10%	0.3	8.7%	X yes □ no
			0.5	4.9%	
			0.7	7.1%	

	Coefficient of variation	≤8%	0.3	5.6%	X yes □ no
			0.5	3.8%	
			0.7	4.0%	

Linearity	Correlation	≥0.9	1,000		X yes □ no

	Linear regression	n.a.	*y* = 0.973*x* + 0.002		X yes □ no

Range	Cell fraction	0.04–1	0.04–1		X yes □ no

Accuracy	Deviation from actual value	0.3 ± 0.03	0.3	0.022	X yes □ no
		0.5 ± 0.05	0.5	0.019	
		0.7 ± 0.07	0.7	0.014	

	Recovery	100 ± 10%	0.3	92.8%	X yes □ no
			0.5	96.1%	
			0.7	98.1%	

Limit of detection	Quantification limit (QL) (10,000 cells)	≤0.04	0.016		X yes □ no

Limit of detection CD8	QL (300,000 cells)	≤0.001	0.001		X yes □ no

Specificity	Fluorescence in channel 1 or 2	Meets specificity	Meets specificity for all antibodies tested		X yes □ no

Efficiency of antibodies	Fraction of positive cells	Within range of development	All antibodies within range		X yes □ no

*Positive cells and negative cells were mixed at the indicated fractions 0.3, 0.5, and 0.7 to represent 30, 50, and 70% of the final cell mixture. For reproducibility, triplicate analysis of three different fractions of positive cells was statistically analyzed by coefficient of variation. The analysis of a different person on a different day was used to determine the intermediate precision. To determine linearity, 10 different fractions of positive cells were analyzed by correlation factor and linear regression. These results were compared with the theoretical real fraction contents to determine accuracy. The limits of detection were determined by analyzing six negative cell fractions and calculated with the formula: 10*s*/*a* (*s* indicating SD and *a* indicating slope of regression). Antibody specificity and efficiency were determined by using positive and negative cell fractions, respectively*.

#### Assessment of Treg Drug Product Purity

Regulatory T cell drug product purity was determined after anti-CD3/anti-CD28 expander bead removal by staining with directly conjugated mouse antihuman IgG_3,κ_ Labeling Check PE (clone AC146, Miltenyi Biotec) and APC (clone AC146, Miltenyi Biotec). Samples were acquired using a FACS Calibur (BD Biosciences), and data were analyzed using CellQuest™ Pro (BD Biosciences) software.

The method to measure anti-CD3/anti-CD28 expander bead and CD25-, CD8- and CD19-labeling bead contamination was validated since it is not described in a Ph. Eur. Specifically, thawed lymphocytes were mixed with predefined amounts of anti-CD3/anti-CD28 expander beads and incubated for 1 h at 37°C at 5% CO_2_. After 1 h of incubation, cells were lysed using saponin 0.2% (Sigma-Aldrich, St. Louis, MI, USA) to also capture potential intracellular beads. Subsequently, beads were stained by flow cytometry with Labeling Check Reagent PE and Labeling Check Reagent APC as described above. To determine bead contamination, all samples were acquired with the use of Trucount^®^ tubes (BD Biosciences) to assure standardized acquisition. Nominal samples negative for beads should show less than 400 beads, nominal samples positive for beads should show more than 400 beads. At least 100 × 10^6^ Treg drug product cells should be lysed, labeled with Labeling Check Reagent PE and Labeling Check Reagent APC and subsequently measured to define bead contamination in the final Treg drug product. The results of the validation are summarized in Table 2.

**Table 2:** Validation results of analysis of anti-CD3/anti-CD28 bead contamination.

Validation part	Parameter	Acceptance	Result		Passed
Reproducibility	Coefficient of variation	≤25	750 1,000 3,000	7.1 8.7 22.7	X yes □ no

Intermediate precision	*F*-value	<9.28	750 1,000 3,000	1.54 5.50 2.49	X yes □ no

	Deviation from mean	≤15%	750 1,000 3,000	6.5 14.1 7.3	X yes □ no

	Coefficient of variation	≤25	750 1,000 3,000	14.0 13.3 17.6	X yes □ no

Linearity	Coefficient of correlation	≤0.98	0.996		X yes □ no

Range	Detection range	300–6,000	300–6,000		X yes □ no

Accuracy	Mean	637–863 850–1,150 2,400–3,600	750 1,000 3,000	689 1,105 3,420	X yes □ no

	Retrieval rate	±15% ±15% ±20%	750 1,000 3,000	8.1% 10.5% 14.0%	X yes □ no

Limit of detection	QL	<400	390		X yes □ no

Specificity	Positive particle	>400	1,105		X yes □ no

	Negative particle	<400	38		X yes □ no

*A total of 750, 1,000, and 3,000 anti-CD3/anti-CD28 expander beads were mixed with 100 × 10^6^ peripheral blood mononuclear cells. For reproducibility, triplicate analysis of indicated cell-bead mixtures was statistically analyzed by coefficient of variation. The analysis of a different person on a different day was used to determine the intermediate precision. To determine linearity, 10 different cell-bead mixtures were analyzed by correlation factor and linear regression. These results were compared with the theoretical real fraction contents to determine accuracy. The limits of detection were determined by analyzing six bead-negative cell mixtures and calculated with the formula: 10*s*/*a* (*s* indicating SD and *a* indicating slope of regression). Specificity was determined by using bead-positive and bead-negative cell mixtures, respectively*.

*QL, quantification limit*.

#### Assessment of Treg Drug Product Function

The method to assess Treg drug product function was validated, since it is not described in the Ph. Eur. Specifically, cryopreserved autologous CD25^−^ cells were thawed, washed, and labeled with 5 µM carboxyfluorescein succinimidyl ester (CFSE) (ThermoFisher Scientific, Carlsbad, CA, USA). Next, CFSE-labeled CD25^−^ cells (containing CD4^+^ and CD8^+^ cells; hereafter called responder cells) were cocultured with thawed day 21 Treg drug product cells (hereafter called Treg) at a Treg to responder cell ratio of 1 + 1, 1 + 5, and 1 + 10. Cocultures were stimulated with anti-CD3 and anti-CD28 coated beads (MACS GMP ExpAct Treg Kit, Miltenyi Biotec) at a bead-to-cell ratio of 1 + 1. Negative controls included responder cells alone and Treg + responders cells at a ratio of 1 + 1 without the addition of anti-CD3 and anti-CD28 coated beads. The positive control included responder cells alone with the addition of anti-CD3 and anti-CD28 coated beads at a bead + responder cell ratio of 1 + 4. The absolute cell concentration and cell density at the beginning of the coculture was 1 × 10^6^/ml and 1 × 10^6^/cm^2^ per well, respectively.

Cocultures were routinely performed in triplicates in 48-well plates and harvested after 60–72 h of incubation at 37 ± 1°C, 5 ± 1% CO_2_, >70% r.h. After 60–72 h of incubation, conditions were harvested, stained with PI and CD8 as described under flow cytometry and acquired using a FACS Calibur (BD Biosciences). Data were analyzed using CellQuest™ Pro (BD Biosciences) software. Treg-mediated suppression was calculated based on the percentage of divided cells in the first cell generation with the positive control set to 100%. One cell generation was defined to contain at least ≥10% divided cells. Based on all cell generations, negative control cells should show less than 5% proliferated cells, and positive control cells should show more than 30% proliferated cells. Lot-release is based on the amount of Treg-mediated suppression in cocultures with a Treg to responder cell ratio of 1 + 1, 1 + 5, and 1 + 10, respectively. For validation, more Treg to responder cell ratios were included. The results of the validation are summarized in Table 3.

**Table 3:** Validation results of analysis of regulatory T cell (Treg)-mediated suppression.

Validation part	Sample	Specificity	Result	Passed
**Reproducibility**				
Coefficient of variation	2 + 1	≤5%	0.3	X yes □ no
	5 + 1	≤5%	1.0	X yes □ no
	10 + 1	≤5%	2.2	X yes □ no
	30 + 1	≤5%	4.5	X yes □ no
	50 + 1	≤10%	4.9	X yes □ no

**Intermediate precision**				
Deviation from mean	2 + 1	≤±8%	5.0	X yes □ no
	5 + 1	≤±12%	7.2	X yes □ no
	10 + 1	≤±20%	12.5	X yes □ no
	30 + 1	≤±30%	−5.6	X yes □ no
	50 + 1	≤±40%	−39.8	X yes □ no

Coefficient of variation	2 + 1	≤10%	2.8	X yes □ no
	5 + 1	≤10%	3.8	X yes □ no
	10 + 1	≤10%	5.4	X yes □ no
	30 + 1	≤15%	4.8	X yes □ no
	50 + 1	≤15%	8.0	X yes □ no

**Linearity**				
Coefficient of correlation in ascending range	100 + 1 to 5 + 1	≥0.8	0.908	X yes □ no

Range	All samples	≤10–≥90%	2.4–97.7%	X yes □ no

**Accuracy**				
Retrieval rate	1 + 1	100 ± 5%	99.7%	X yes □ no
	5 + 1	100 ± 5%	98.4%	X yes □ no
	10 + 1	100 ± 10%	108.8%	X yes □ no
**Limit of detection**				
QL proliferation		≤1%	0.4%	X yes □ no
QL suppression		≥95%	99.99%	X yes □ no

*Responder cells and Treg were mixed at the indicated ratios of 2 + 1, 5 + 1, 10 + 1, 30 + 1, and 50 + 1. For reproducibility, triplicate analysis of responder cell to Treg ratios was statistically analyzed by coefficient of variation. The analysis of a different person on a different day was used to determine the intermediate precision. To determine linearity, 10 different responder cell to Treg ratios (1,000 + 1, 200 + 1, 100 + 1, 50 + 1, 30 + 1, 10 + 1, 5 + 1, 2 + 1, 1 + 1, and 1 + 2) were analyzed by correlation factor and linear regression. These results were compared with the theoretical real fraction contents to determine accuracy. The limits of detection were determined by analyzing six non-stimulated responder cell to Treg mixtures (1 + 1) and calculated with the formula: 10*s*/*a* (*s* indicating SD and *a* indicating slope of regression)*.

*QL, quantification limit*.

#### Assessment of the Treg Drug Product Concerning Viability, Cell Number, Cell Concentration, Sterility, Bacterial Endotoxins, and Mycoplasma DNA

Cell number, cell concentration, and cell viability are determined by trypan blue staining and microscopic examination using a hemocytometer according to the method described in Ph. Eur. 2.7.29. Sterility testing is routinely performed according to Ph. Eur. 2.6.1 by Bioservice Scientific Laboratories (BSL) GmbH, Planegg, Germany. Testing on bacterial endotoxins is routinely performed according to Ph. Eur. 2.6.14 by BSL. Testing on mycoplasma DNA is achieved by PCR by an in-house validated method by BSL.

### T Cell Receptor (TCR) Vβ Repertoire Analysis

The TCR Vβ repertoire of *ex vivo* generated CD25^+^ cells was determined by using the IO Test Beta Mark TCR Vβ Repertoire kit (Beckman Coulter, France) as previously published (28). Day 0 derived CD25^+^ cells and day 21 CD25^+^ cells were stained and analyzed for TCR Vβ specificity according to the manufacturer’s instructions.

### Epigenetic Analysis

Genomic cellular DNA was isolated using a high pure PCR template preparation kit (Roche). Next, sodium bisulfite conversion of the purified DNA was performed by using the EpiTect^®^ Fast DNA Bisulfite Kit (Qiagen) according to the manufacturer’s instructions. The following primers and probe enabled us to specifically detect methylated FoxP3 (29):

5′-TGTCGATGAAGTTCGGCGTAT-3′ (forward)
5′-CCCCCGACTTACCCAAATTT-3′ (reverse)
6FAM-5′-CGGTCGTTATGACGTTAATGGCGGA-3′-TAMRA (probe)
Primers and probes for the detection of unmethylated FoxP3 were designed accordingly:
5′-TGTTGATGAAGTTTGGTGTAT-3′ (forward)
5′-CCCCCAACTTACCCAAATTT-3′ (reverse)
6FAM-5′-TGGTTGTTATGATGTTAATGGTGGA-3′-TAMRA (probe).

Quantitative PCR (qPCR) was performed by using the maxima probe qPCR master mix (ThermoFisher Scientific) and a C1000TM Thermal Cycler (Bio-Rad). Percentage of methylation in cells was calculated as Meth. [%] = 100/[1 + 2^ΔCt(meth–unmeth)^]% as recently described (30). ΔCt(meth − unmeth) represents the difference between the Ct value of methylated FoxP3 signal and Ct value of unmethylated FoxP3 value. Hypomethylation was calculated as hypometh. [%] = 100% − meth. [%]. Since the male FoxP3 Treg-specific demethylated region (TSDR) is described to be fully demethylated, and the female TSDR shows hemimethylation (31), the above described calculation would underestimate the relative number of hypomethylated Treg in cells derived from human donors. To circumvent an underestimation, the methylation index for female-derived probes was corrected using the following formula: meth. [%]_female_ = meth. [%] − (100 − meth. [%]). Similarly, hypomethylation in female derived probes was calculated as hypometh. [%] = 100% − meth. [%]_female_.

### Assessment of Homing Markers

The homing potential of day 0 CD25^+^ cells and day 21 Treg drug product cells was assessed by staining with directly conjugated mAbs against CCR4 (clone L21H4), CCR8 (clone 191704), CD62L (clone DREG-56), CD103 (clone Ber-ATC8), CXCR3 (clone G025H7), PSGL-1 (clone 688101), CCR9 (clone L053E8), CCR5 (clone HEK/1/85a), alpha4 Integrin (clone MZ18-24A9), beta7 Integrin (clone FIB27), and the purified mAb GPR15 (clone 367902) with subsequent staining with specific mouse secondary IgG2b APC labeled antibody. Corresponding mouse isotype controls were included to assess unspecific binding. Cells were acquired using an LSR Fortessa (BD Biosciences), and data were analyzed using FlowJo software.

### Statistical Analysis

Statistical differences as measured by a two-sided paired Student *t* test were calculated using Excel v2010, based on the number of experiments as indicated in the figure legends. Differences were considered to be significant at a *P* value less than 0.05.

## Results

### Treg Drug Product Expansion, Viability, and Cell Number

**Figure 2:**
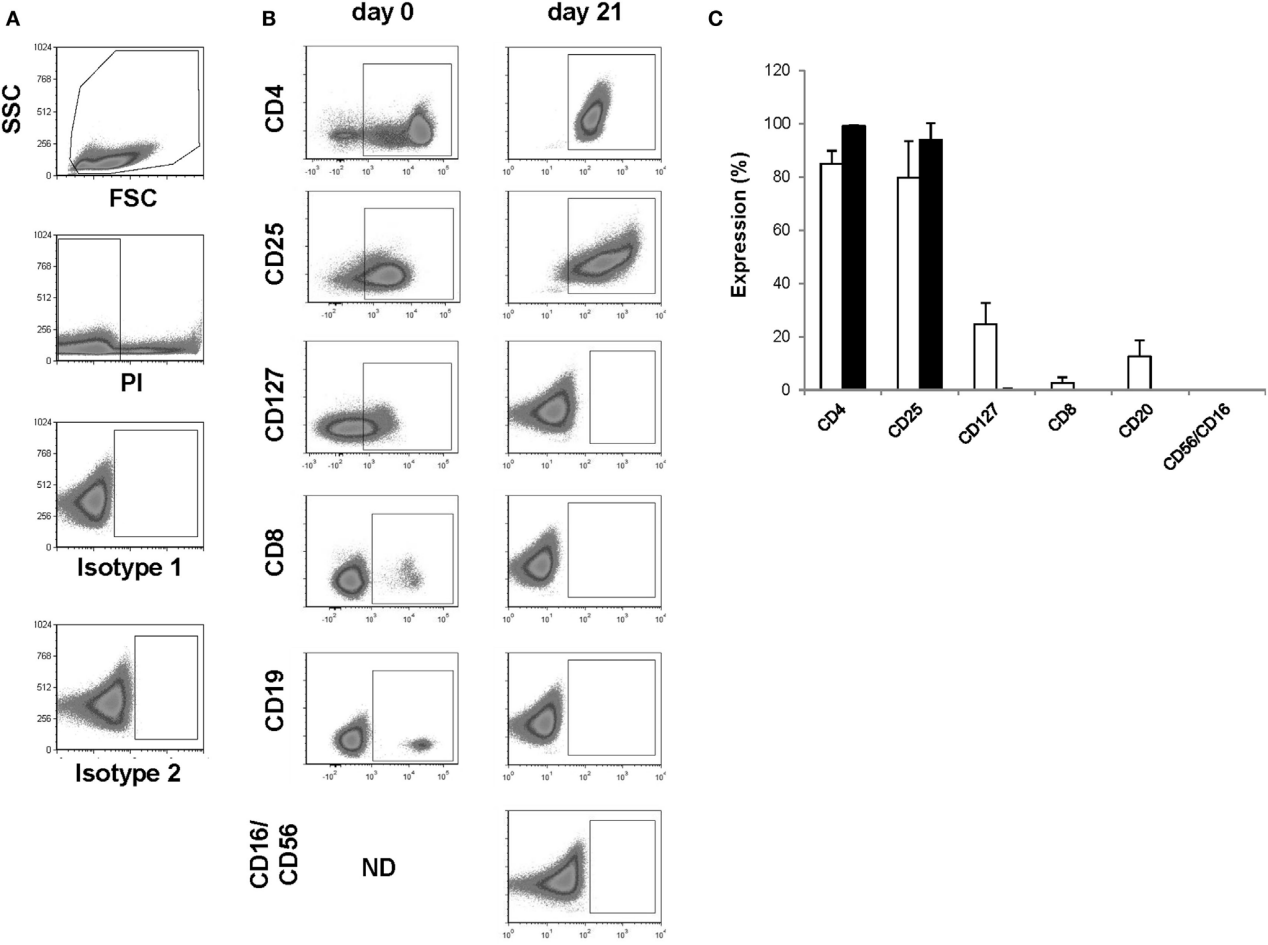
Assessment of identity and cellular composition. **(A)** Representative FACS plots showing the gating strategy. **(B)** Representative FACS plots gated on PI^−^ cells showing CD4, CD25, CD127, CD8, CD19, and CD56/CD16 expression after CD25^+^ cell enrichment at day 0 and after thawing the day 21 regulatory T cell (Treg) drug product. **(C)** Proportion of PI^−^ cells expressing CD4, CD25, CD127, CD8, CD19, and CD56/CD16 after CD25^+^ cell enrichment at day 0 (*n* = 4, white bars) and after thawing the day 21 Treg drug product (*n* = 4, filled bars). ND, not determined.

To validate the established GMP-complaint production protocol and the predefined lot-release criteria, we performed four subsequent Treg productions with a healthy donor-derived leukapheresis product. Such a production is also called “consistency run” (hereafter called Con) and is executed by exactly following the predefined protocol and predefined lot-release criteria. A minimum of three consecutive Cons who pass all predefined lot-release criteria is required to obtain official GMP production approval from the German authorities. With use of the protocol, leukapheresis-derived CD25^+^ cells expanded greater than 2 orders of magnitude with an average cell number of 113 × 10^6^ at day 7 (range 34 × 10^6^–199 × 10^6^), 501 × 10^6^ at day 11 (range 158 × 10^6^–849 × 10^6^), 635 × 10^6^ at day 14 (range 234 × 10^6^–1,020 × 10^6^), 986 × 10^6^ at day 18 (range 159 × 10^6^–2,074 × 10^6^), and 1,076 × 10^6^ (range 528 × 10^6^–1,440 × 10^6^) at day 21 after anti-CD3/anti-CD28 expander bead removal (Figures 2A,B). The exact starting Treg number and exact expansion rate per consistency run are shown in File S1 in Supplementary Material. In addition, cell viability met the predefined limit of ≥75% viable cells at day 7 (range 94.7–98.3%), day 11 (range 93.5–98.7%), day 14 (range 95.8–97.6%), day 18 (range 94.3–98.8%), and day 21 (range 92.9–98.3%) after anti-CD3/anti-CD28 expander bead removal in every consistency run (Table 4).

**Table 4:** Good manufacturing practice-compliant production process evaluation.

Parameter	Limit	Con1	Con2	Con3	Con4
Viable cells/ml	≥5.0 × 10^6^	10.5 × 10^6^	6.3 × 10^6^	10.8 × 10^6^	8.2 × 10^6^
Cell viability (%)	≥75	96.7	92.8	96.8	96.9
Sterility	No growth	No growth	No growth	No growth	No growth
Endotoxin (IU/ml)	≤30	≤30	≤30	≤30	≤30
Mycoplasma	Negative	Negative	Negative	Negative	Negative
CD4 (%)	≥90.0	99.6	99.2	98.6	99.4
CD25 (%)	≥80.0	98.9	98.6	85.5	93.3
CD127 (%)	≥10.0	0.00	0.05	0.68	0.00
CD8 (%)	≤0.10	0.00	0.00	0.06	0.00
CD19 (%)	≤1.0	0.00	0.00	0.00	0.00
CD56/16 (%)	≤1.0	0.00	0.00	0.06	0.00
Labeling check reagent PE (%)	≤400	≤400	≤400	≤400	≤400
Labeling check reagent APC (%)	≤400	≤400	≤400	≤400	≤400
Suppression at ratio 1 + 1 (%)	≥80.0	99.5	81.4	98.2	99.9
Suppression at ratio 1 + 5 (%)	≥60.0	96.1	60.4	97.2	99.4
Suppression at ratio 1 + 10 (%)	≥50.0	85.4	52.5	61.6	83.2

*Four successive regulatory T cell (Treg) productions were performed with healthy donor-derived leukapheresis products. Productions passed the indicated limits for release. Viable cells per milliliter indicate the total number of life retrieved Treg in the final Treg product. Viability indicates the percentage of life cells in the final Treg product. Sterility indicates bacterial growth contaminating the final Treg product. CD4, CD25, CD127, CD8, CD19, and CD56/CD16 indicate surface expression on the final Treg product. Labeling check reagent PE and APC indicate the remaining number of anti-CD3/anti-CD28 beads in the final Treg product. Suppression at ratio 1 + 1, 1 + 5, and 1 + 10 indicate the amount of suppression within the first generation of proliferating CD8^+^ responder cells by the final Treg product*.

### Treg Drug Product Sterility, Bacterial Endotoxins, and Mycoplasma DNA

One thawed vial of the day 21 Treg drug product from each consistency run was tested for its sterility and the presence of bacterial toxins and mycoplasma DNA. As confirmed by BSL, no bacterial growth was contaminating the Treg drug product after 21 days of expansion. Also, no bacterial endotoxins and no mycoplasma DNA could be detected (Table 4).

### Treg Drug Product Identity and Cellular Composition

The presence of CD4 (≥90.0%) and CD25 (≥80.0%) in combination with low to absent surface expression of CD127 (≤10.0%) is used to phenotypically discriminate Treg from effector T cells. This approach has been elected based on studies by Liu et al. who reported that CD127 surface expression inversely correlates with FoxP3 and suppressive function of human CD4^+^ Treg^3^. A cutoff of more than 80.0 and 90.0% was chosen for CD25 and CD4, respectively. A cutoff of less than 10.0% was chosen for CD127. Moreover, the Treg drug product cellular composition is guaranteed by the stringent cutoff criteria for potential CD8 (≤0.1%), CD19 (≤1.0%), and CD56/CD16 (≤1.0%) cells contaminating the Treg drug product. These cutoff criteria were based on the results of several clinical studies in our department with dendritic cells (32, 33). In these studies, a cellular contamination, based on the sum of CD3^+^ T cells, CD19^+^ B cells, and CD56^+^ NK-cells, of up to 10% of the total infused cells was tolerated well by patients with cutaneous or ocular melanoma. In the case of *ex vivo* expanded autologous Treg, contamination with CD8^+^ T cells potentially induces inflammation in patients with autoimmune disorders. Therefore, a cutoff of less than 0.1% contaminating CD8^+^ cells was chosen for the release of the Treg drug product and a cutoff of less than 1.0% was chosen for contaminating CD19 and CD56/CD16 cells. Importantly, to reliably show less than 0.1% CD8^+^ cell contamination in the final Treg product, a total of 300,000 cells must be acquired by flow cytometry. As shown in Figure 2 and Table 4, the performed consistency runs passed the predefined lot-release criteria for product identity and cellular composition by the validated flow cytometry method. Specifically, an average of 99.2% CD4 (range 98.6–99.6%), 94.1% CD25 (range 85.5–98.9%), 0.18% CD127 (range 0.0–0.68%), 0.02% CD8 (range 0.0–0.06%), 0.0% CD19 (range 0.0–0.0%), and 0.2% CD56/16 (range 0.0–0.06%) was determined in the thawed Treg drug products. In addition, as an internal scientific in-process control, intracellular FoxP3 expression was determined on enriched day 0 CD25^+^ cells and day 21 expanded CD25^+^ cells (File S2 in Supplementary Material).

### Treg Drug Product Purity

**Figure 3:**
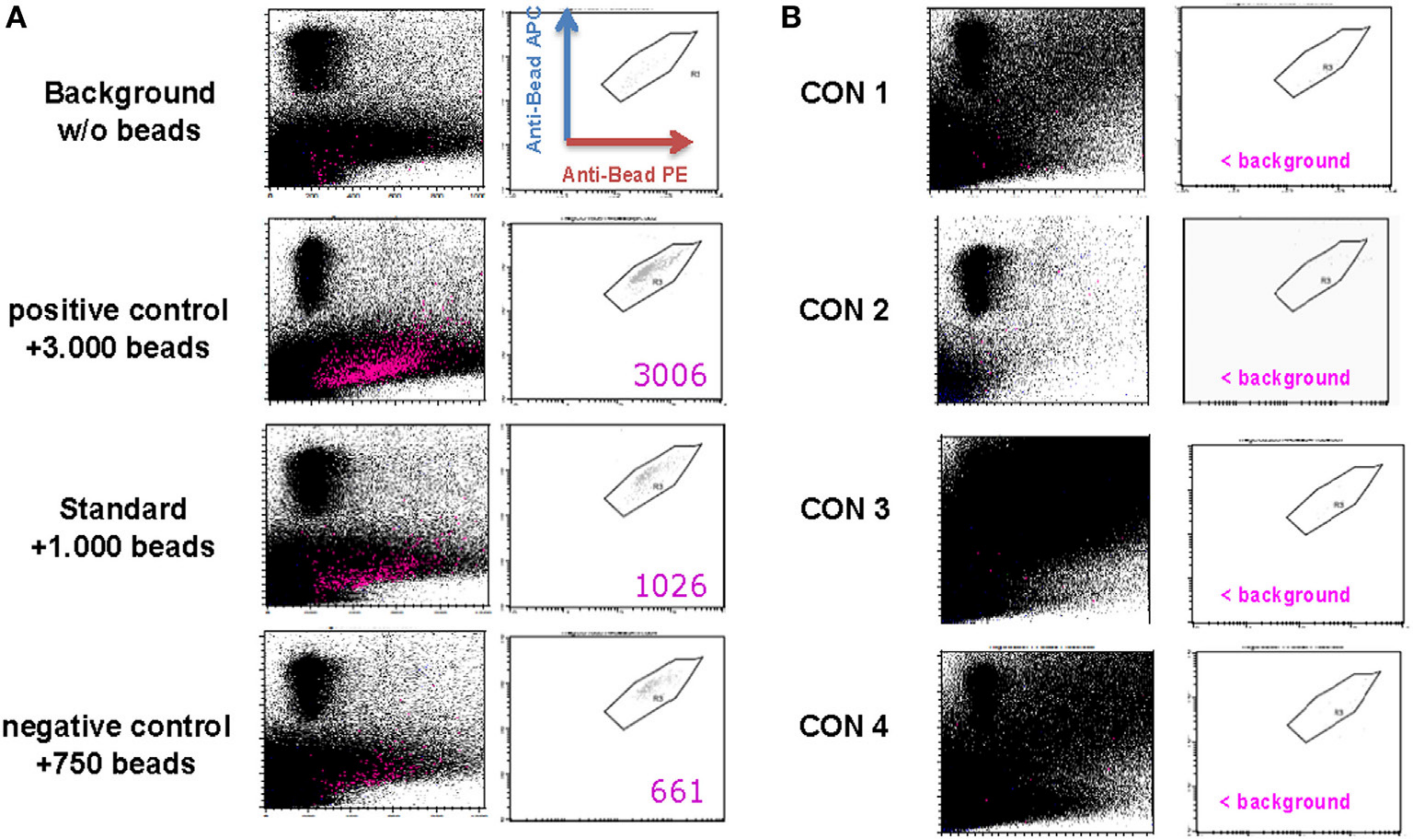
Assessment of regulatory T cell (Treg) drug product purity. **(A)** Representative FACS plots gated on PE^+^/APC^+^ anti-CD3/anti-CD28 expander beads in a control sample containing no anti-CD3/anti-CD28 expander beads (=background sample), a control sample containing 750 anti-CD3/anti-CD28 expander beads (=negative control sample), a control sample containing 1,000 anti-CD3/anti-CD28 expander beads (=standard sample), and a control sampler containing 3,000 anti-CD3/anti-CD28 expander beads. **(B)** FACS plots gated on PE^+^/APC^+^ anti-CD3/anti-CD28 expander beads in a thawed Treg drug product from consistency run 1 (=Con1), consistency run 2 (=Con2), consistency run 3 (=Con3), and consistency run 4 (=Con4).

The anti-CD3/anti-CD28 expander beads are removed from the cell product after 21 days of expansion using the CliniMACS^®^ system. Although the CD25-, CD8-, and CD19-labeling beads generally are metabolized during the expansion process, potential remaining labeling beads will simultaneously be removed from the Treg drug product. As defined by our in-house validated bead removal method and shown in Figure 3 and Table 4, bead removal was efficient, since in a total of 100 × 10^6^ lysed Treg product cells less than 400 beads could be retrieved by flow cytometry.

### Treg Drug Product Function

**Figure 4:**
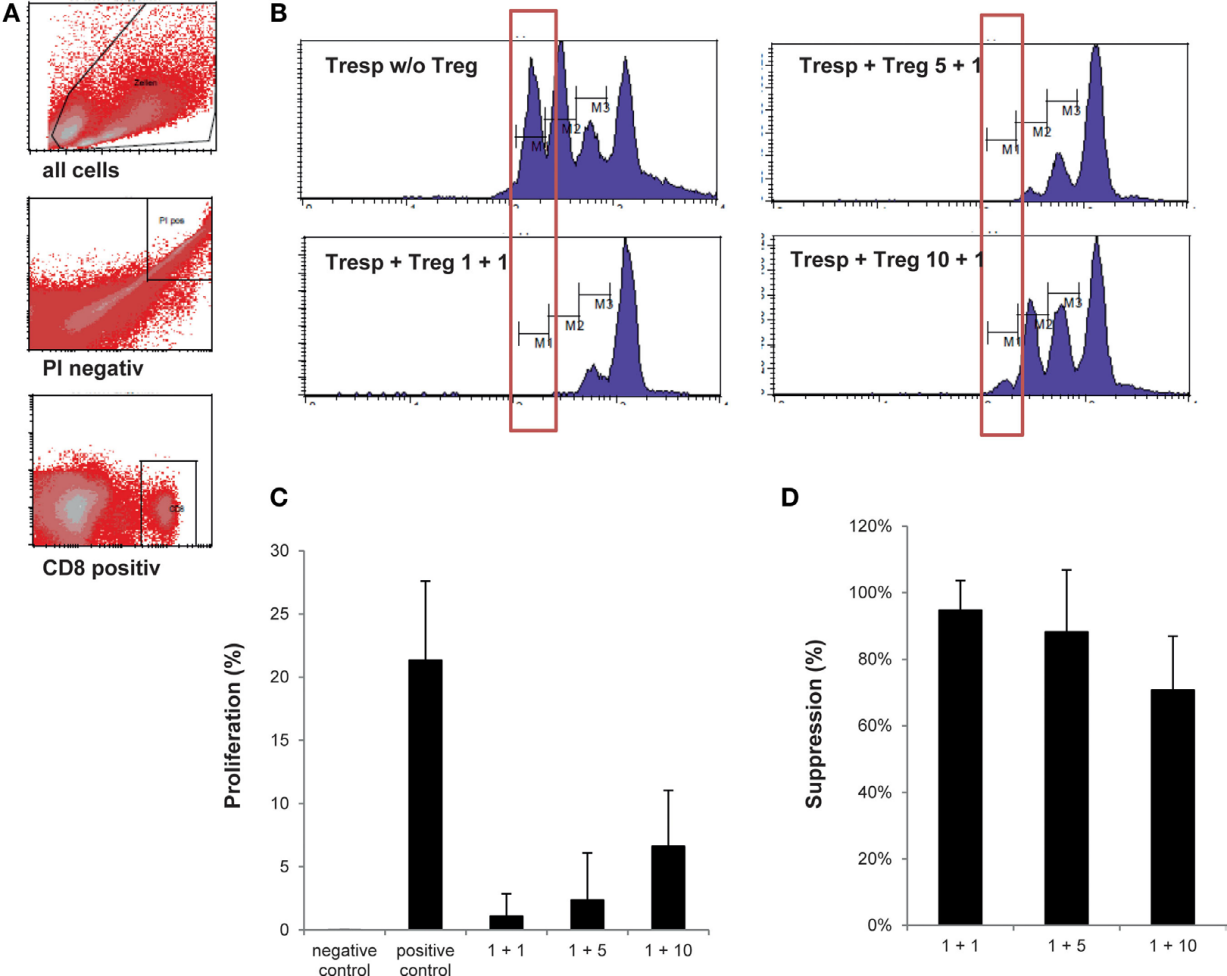
Assessment of regulatory T cell (Treg) drug product function. **(A)** Representative FACS plots showing the gating strategy defining CD25^−^ responder cell proliferation. **(B)** Representative histograms gated on CD8^+^/CFSE^+^ responder cells showing percentage of responder cell proliferation within the most divided cell generation in the presence of the thawed day 21 Treg drug product cells at a Treg to responder cell ratio of 1 + 1, 1 + 5, and 1 + 10, respectively. **(C)** Proportion of the first generation of responder cells (*n* = 4) showing proliferation in the presence of no anti-CD3/anti-CD28 beads (=negative control), in the presence of anti-CD3/anti-CD28 beads (=positive control) and at a Treg to responder cell ratio of 1 + 1 (mean 1.09%), 1 + 5 (mean 2.37%), and 1 + 10 (mean 6.62%), respectively. **(D)** Amount of suppression in the first generation of divided cells at a Treg to responder cell ratio of 1 + 1 (mean 94.7%), 1 + 5 (mean 88.3%), and 1 + 10 (mean 70.7%).

Currently, the intracellular expression of FoxP3 is the most recognized marker to define Treg in human, yet intracellular FoxP3 staining mostly shows high intra-sample variation. In addition, consensus on which antibody clone to include in staining protocols is inconclusive (32–34). We, therefore, elected to omit FoxP3 expression as a separate lot-release criterion. To compensate for the lack of a highly specific Treg marker as part of our lot-release criteria, we established a biologic assay confirming the suppressive nature of the thawed Treg drug product. The objective was to reach ≥80.0% suppression within the first generation of divided cells at a responder cell to Treg ratio of 1 + 1, ≥60.0% suppression within the first generation of divided cells at a responder cell to Treg ratio of 1 + 5, and ≥50.0% suppression within the first generation of divided cells at a responder cell to Treg ratio of 1 + 10. In addition, suppression within the first generation of divided cells at a responder cell to Treg ratio of 1 + 1 had to exceed suppression within the first generation of divided cells at a responder cell to Treg ratio of 1 + 5. Likewise, suppression within the first generation of divided cells at a responder cell to Treg ratio of 1 + 5 had to exceed suppression within the first generation of divided cells at a responder cell to Treg ratio of 1 + 10. As shown in Figure 4 and Table 4, an average responder cell proliferation of 21.3% was observed within the first generation of divided cells when no Treg were added to the coculture. In the presence of Treg at a ratio of 1 + 1, responder cells showed an average of 1.09% of proliferation within the first generation of divided cells, whereas 2.37 and 6.62% of proliferation was observed at a ratio of 1 + 5 and 1 + 10, respectively (Figure 4C). The Treg-mediated suppression within the first generation of divided cells was calculated with the positive control set to 100%, resulting in an average suppression of 94.7% at a responder cell to Treg ratio of 1 + 1 (range 81.4–99.9%), 88.3% suppression at a responder cell to Treg ratio of 1 + 5 (range 60.4–99.3%) and 70.7% suppression at a responder cell to Treg ratio of 1 + 10 (range 52.5–85.5%) (Figure 4D). In addition, average suppression within the first generation of divided cells at a responder cell to Treg ratio of 1 + 1 exceeded suppression within the first generation of divided cells at a responder to Treg ratio of 1 + 5. Likewise, average suppression within the first generation of divided cells at a responder cell to Treg ratio of 1 + 5 exceeded suppression within the first generation of divided cells at a responder to Treg ratio of 1 + 10. Moreover, based on all proliferated CD8^+^ cells, including all cell generations, a gradual increase in mean fluorescence intensity (MFI) was observed with increasing Treg to responder cell ratios with a mean MFI of 522.8 (range 440–705) at a Treg to responder cell ratio of 1 + 10 and a mean MFI of 667.5 (range 539–893) at a Treg to responder cell ratio of 1 + 1 (File S3A–C in Supplementary Material). Likewise, a reduction in the total number of cell generations was observed with increasing Treg to responder cell ratios (File S3D in Supplementary Material).

### Treg Drug Product Stability

Stability data were acquired with Treg drug products that were continuously stored in the gas phase of liquid nitrogen ≤−150°C for at least 12 months. Stored Treg drug products were thawed and analyzed according to the criteria defined for lot-release. Microbial testing, phenotyping, cellular composition, viability, recovery, and function met the limits of the drug product in every case for up to 12 months (Table 5). Therefore, guaranteed stability for the Treg drug product was set at 12 months.

**Table 5:** Results of regulatory T cell (Treg) drug product stability testing after at least 12 months of storage.

Parameter	Con1		Con2		Con3		Con4		Acceptance criteria	Criteria passed
	Release	>12 months	Release	>12 months	Release	>12 months	Release	>12 months		
CD4 (%)	99.6	99.7	99.2	99.2	98.6	98.7	99.4	99.3	≥90.0	Yes
CD25 (%)	98.9	95.7	98.6	98.3	85.5	81.9	93.3	93.8	≥80.0	Yes
CD127 (%)	0.0	1.4	0.1	2.5	0.7	1.6	0.0	1.2	≤10.0	Yes
CD8 (%)	0.0	0.1	0.0	0.0	0.1	0.1	0.0	0.1	≤0.5	Yes
CD19 (%)	0.0	0.0	0.0	0.0	0.0	0.0	0.0	0.0	≤1.0	Yes
CD56/16 (%)	0.0	0.4	0.0	0.0	0.0	0.0	0.0	0.0	≤1.0	Yes
Suppression at ratio 1 + 1 (%)	99.5	95.2	81.4	99.4	98.2	96.1	99.9	98.4	≥80.0	Yes
Suppression at ratio 5 + 1 (%)	96.1	80.8	60.4	85.9	97.2	66.9	99.4	79.2	≥60.0	Yes
Suppression at ratio 10 + 1 (%)	85.4	66.5	52.5	65.9	61.6	61.8	83.2	70.8	≥50.0	Yes
viable cells/ml (×10^6^)	10.5	9.3	6.3	9.1	10.8	9.8	8.2	7.9	≥5.0	Yes
Cell viability (%)	96.7	78.7	92.8	79.7	96.8	84.1	96.9	74.1	≥50.0	Yes
Sterility	ng	ng	ng	ng	ng	ng	ng	ng	ng	Yes
Endotoxin (IE/ml)	<30	<30	<30	<30	<30	<30	<30	<30	<30	Yes
Mycoplasma	neg	neg	neg	neg	neg	neg	neg	neg	neg	Yes

*Treg were thawed after a minimum of 24 h after production or after at least 12 months of storage at ≤−150°C and analyzed for lot-release. CD4, CD25, CD127, CD8, CD19, and CD56/CD16 indicate surface expression on the final Treg product. Suppression at ratio 1 + 1, 1 + 5, and 1 + 10 indicate the amount of suppression within the first generation of proliferating CD8^+^ responder cells by the final Treg product. Viable cells per milliliter indicate the total number of life retrieved Treg in the final Treg product. Viability indicates the percentage of life cells in the final Treg product. Sterility indicates bacterial growth contaminating the final Treg product*.

*ng, no growth; neg, negative*.

Moreover, Treg drug products stored for at least 12 months from each consistency run were thawed, diluted with 0.9% sodium chloride solution and incubated for 90 min at 30 ± 1°C to mimic the preparation of the Treg drug product for adoptive transfer in clinical settings. After 90 min of incubation at 30 ± 1°C in a 0.9% sodium chloride solution, phenotype, cellular composition, viability, recovery, and function met the preset limits of the drug product in all tested products (Table 6).

**Table 6:** Results of regulatory T cell (Treg) drug product stability testing in the clinical application solution.

Parameter	Con1		Con2		Con3		Con4		Acceptance criteria	Criteria passed
	0.5 × 10^6^/ml	20 × 10^6^/ml	0.5 × 10^6^/ml	20 × 10^6^/ml	0.5 × 10^6^/ml	20 × 10^6^/ml	0.5 × 10^6^/ml	20 × 10^6^/ml		
CD4 (%)	93.2	98.9	97.3	98.2	98.7	98.4	99.5	99.4	≥90.0	Yes
CD25 (%)	89.1	87.5	86.1	96.6	81.1	86.0	95.9	94.6	≥80.0	Yes
CD127 (%)	0.2	0.1	0.9	0.0	6.8	4.8	3.3	1.9	≤10.0	Yes
CD8 (%)	0.0	0.1	0.0	0.0	0.1	0.1	0.0	0.1	≤0.5	Yes
CD19 (%)	0.0	0.0	0.0	0.0	0.0	0.0	0.0	0.0	≤1.0	Yes
CD56/16 (%)	0.0	0.4	0.0	0.0	0.0	0.0	0.0	0.0	≤1.0	Yes
Suppression at ratio 1 + 1 (%)	97.7	96.5	98.8	97.6	97.0	96.5	93.5	96.6	≥80.0	Yes
Suppression at ratio 5 + 1 (%)	90.3	91.7	94.7	96.2	91.7	76.1	82.5	92.6	≥60.0	Yes
Suppression at ratio 10 + 1 (%)	81.6	87.1	70.3	70.3	78.5	63.6	56.9	55.2	≥50.0	Yes
Viable cells/ml (×10^6^)	7.3	6.7	6.1	5.7	6.0	5.5	7.1	5.7	≥5.0	Yes
Viability (%)	76.1	70.5	78.0	70.6	77.2	79.1	74.9	76.3	≥50.0	Yes

*Treg were thawed at least 12 months after storage at ≤−150°C and analyzed for stability in a 0.9% sodium chloride solution at indicated Treg concentrations. CD4, CD25, CD127, CD8, CD19, and CD56/CD16 indicate surface expression on the final Treg product. Suppression at ratio 1 + 1, 1 + 5, and 1 + 10 indicate the amount of suppression within the first generation of proliferating CD8^+^ responder cells by the final Treg product. Viable cells per milliliter indicate the total number of life retrieved Treg in the final Treg product. Viability indicates the percentage of life cells in the final Treg product*.

### Treg Drug Products Are Polyclonal, Hypomethylated, and Express Various Markers Associated with Tissue Homing

**Figure 5:**
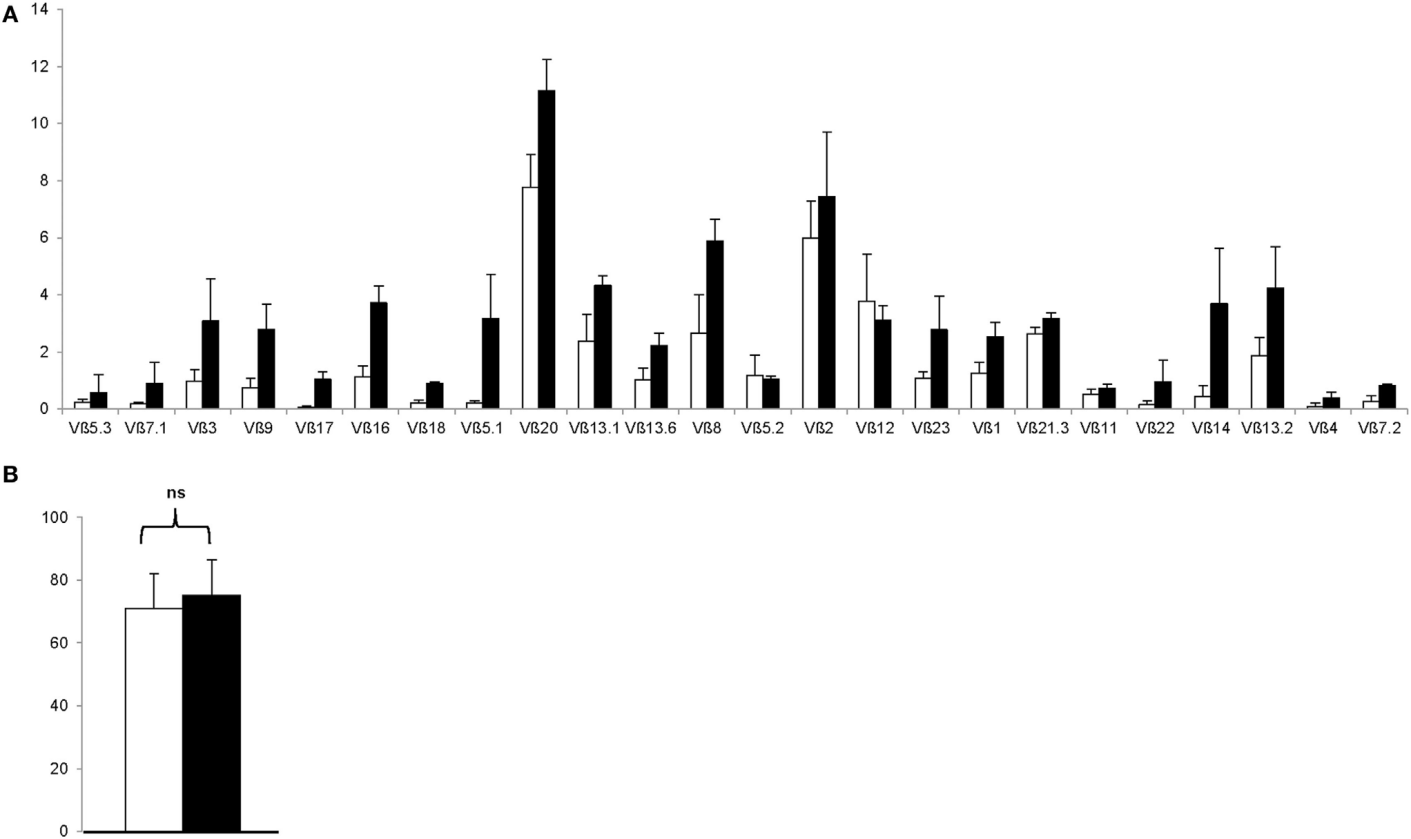
The regulatory T cell (Treg) drug product is polycloncal and hypomethylated at intron 1 of the FoxP3 locus. **(A)** Proportion of Treg expressing indicated T cell receptor Vβ subtype after CD25^+^ cell enrichment at day 0 (*n* = 4, white bars) and after 21 days of *ex vivo* expansion (*n* = 4, filled bars). **(B)** Percentage hypomethylation at intron 1 of the FoxP3 locus after CD25^+^ cell enrichment at day 0 (*n* = 4, white bar) and after 21 days of *ex vivo* expansion (*n* = 4, filled bar). NS, not significant.

To rule out the outgrowth of monoclonal Treg clones during the GMP-compliant production process, Treg drug products were thawed and stained with a panel of 24 distinct TCR Vβ monoclonal antibodies, which cover approximately 70% of the human TCR Vβ repertoire. As shown in Figure 5A, both enriched day 0 CD25^+^ cells and day 21 Treg expressed all 24 TCRs, indicating that the produced Treg remain polyclonal. In addition, GMP-compliant Treg production does not affect hypomethylation of the Treg at intron 1 of the FoxP3 locus, since no significant difference in hypomethylation was found between enriched day 0 CD25^+^ cells and day 21 Treg drug products (*P* = 0.5111, Figure 5B).

**Figure 6:**
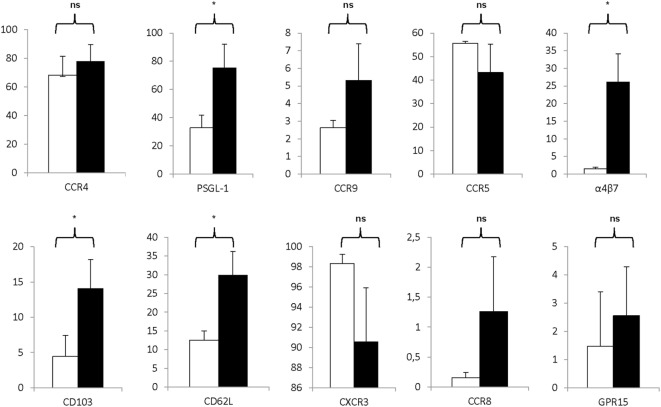
Markers associated with homing are significantly expressed on the regulatory T cell (Treg) drug product. Proportion of CD25^+^ cells at day 0 (white bars) and day 21 Treg drug product cells (filled bars) expressing CCR4 (*n* = 4), PSG-1 (*n* = 4), CCR9 (*n* = 4), CCR5 (*n* = 4), α4β7 (*n* = 4), CD103 (*n* = 4), CD62L (*n* = 4), CXCR3 (*n* = 4), CCR8 (*n* = 4), and GPR15 (*n* = 4). NS, not significant, *P < 0.05.

Moreover, as shown in Figure 6, the Treg drug product expressed various receptors associated with lymphocyte trafficking into tissues and to sites of inflammation. Specifically, expanded Treg expressed moderate to high levels of PSGL-1, α4β7 integrin, CD103, CCR4, and CD62L and expression levels significantly increased during the expansion process with *P* = 0.0022 for PSGL-1, *P* = 0.0073 for α4β7 integrin, *P* = 0.0138 for CD103, *P* = 0.0248 for CCR4, and *P* = 0.0036 for CD62L. Also, high levels of CCR5 and CXCR3 were expressed on both day 0 CD25^+^ enriched cells and day 21 Treg, whereas only little to moderate expression levels of CCR8, CCR9, and GPR15 were detected.

### Risk Management and Limits of Detection

A major risk in the presented protocol is the detection of human immunodeficiency virus (HIV), hepatitis B virus (HBV), or hepatitis C virus (HCV) antigens or microbiological impurities in the patient material or final Treg product. When the leucapherisate is tested positive for HIV, HBV, or HCV, the production process is stopped immediately, and cell cultures will be destroyed accordingly. When the final drug product is tested positive for microbiological impurities, the product will be placed into quarantine, and is not released for clinical treatment. Confirmation tests will be performed to define if the product remains positive, and will be destroyed or was tested false positive.

Other Treg production risks include a low number of Treg after 21 days of expansion. In this case, the amount of cells regularly used for lot-release (120 × 10^6^) could be reduced accordingly. Any out of specification result for Treg phenotype or Treg function results in the non-release of the Treg product. However, if the number of CD8^+^ cells, CD19^+^ cells, and/or anti-CD3/anti-CD28 expander beads exceeds the specification, the CD8^+^/CD19^+^ cell depletion and/or anti-CD3/anti-CD28 expander bead removal step could be repeated. Applicable limits of detection and quantification are provided in Tables 1–3, respectively.

## Discussion

Here, we show, for the first time, an official authority GMP-approved protocol to produce large numbers of *ex vivo* rapamycin-expanded CD25^+^ cells intended to treat inflammatory and autoimmune disorders. In addition, we provide the complete testing and validation of the lot-release of the final Treg drug product after freezing and thawing. Moreover, we extended traditional lot-release criteria and added a functional biological assay assuring the suppressive nature of the produced Treg cells at different Treg-to-effector T cell ratios. Thus far, published clinical studies testing adoptively transferred Treg included classical suppression assays as part of the immune-monitoring assays (18) after treatment of the patients to correlate clinical outcome with *in vitro* Treg function, but the delivered Treg product did not undergo potency testing before administration to the patient. By contrast, this Treg product is not released for clinical treatment unless *in vitro* suppression is proven at various cell ratios for each batch of Treg.

All the consistency runs met the specifications of the process and the product, including sterility, Treg phenotype, non-Treg cellular contamination, anti-CD3/anti-CD28 expander bead purity, and Treg function after freezing and thawing. This contrasts the general consideration that cryopreservation of Treg products is challenging (35) and that stimulation and expansion steps are necessary to restore Treg function after thawing (36).

Stability data were acquired with Treg, which were continuously stored in the gas phase of liquid nitrogen at ≤150°C for at least 12 months. Stored Treg were shown to retain Treg phenotype and function. Therefore, as advocated by Singer et al. (12), our Treg drug product could facilitate an “on demand” treatment for an acute inflammatory disease or acute allograft rejection without the time delay required for Treg enrichment and expansion. Moreover, since stability is warranted for at least 12 months, multiple Treg doses could be administered to the patient at different time points.

Besides Treg drug product stability after freezing and thawing, the Treg also remained stable after dilution in a 0.9% physiological saline infusion solution for up to 90 min. This is an important assurance, since the Treg drug product will be transferred intravenously through continuous infusion using 50 ml syringes in a perfusion pump.

Importantly, this Treg expansion protocol has several differences compared with the Treg expansion protocol intended to treat patients after liver transplantation (27). First, the presented production protocol reaches clinically relevant Treg numbers after 21 days of expansion without the need to re-stimulate the expanding Treg. Second, less than 0.1% of contaminating CD8^+^ effector cells were present in the released Treg product. This is a fraction of the allowed 10% CD8^+^ effector cells in the Treg product intended to prevent liver rejection. Third, expanded Treg remain hypomethylated at intron 1 of the FoxP3 locus, confirming their epigenetic stability. Finally, the produced Treg show suppressive function against autologous CD8^+^ effector cells at various Treg-to-effector cell ratios; whereas the Treg produced to prevent liver rejection showed suppression of allogeneic effector cells at one Treg-to-effector cell ratio.

In the past years, several clinical studies employing expanded Treg have been conducted. The majority of studies included patients at risk for GvHD (14–16) or organ rejection (37, 38) after transplantation. These studies either infused *ex vivo* expanded Treg (14–16) or freshly isolated non-expanded Treg cells (39, 40). Treg infusions were well tolerated and no dose-limiting toxicities were reported (14, 15). In addition, the onset of both acute and chronic GvHD was favorably affected compared with historical controls and no adverse effects on non-relapse mortality or relapse were detected within a minimum follow-up of 2 years (14, 15). In addition, the possibility of adoptive Treg to ameliorate insulin dependency in both children and adults has been reported (17, 18). In these studies, Treg transfer was safe and not associated with serious adverse events in the treated children (17). By contrast, four serious adverse events were reported in the treated adults (18). Specifically, one patient experienced three episodes of serious hypoglycemia 14, 248, and 463 days after Treg treatment, and one patient experienced an episode of diabetic ketoacidosis 67 days after Treg treatment. Interestingly, by labeling with [6,6-2H2] glucose, Treg were demonstrated to persist in the peripheral circulation for up to one year after transfer (18). Finally, the safety and efficacy of *ex vivo* expanded ovalbumin-specific IL-10-producing Treg has been assessed in patients with Crohn’s disease (CD). The safety profile in this pilot study showed good tolerability and adverse events reflected the underlying CD. Moreover, a clinical significant improvement of disease symptoms was noted 5 weeks after Treg infusion in 40% of patients (41).

In conclusion, Treg produced by the presented method have broad clinical potential. Based on the fact that the presented Treg drug product is polyclonal and expressing various receptors associated with (i) lymphocyte trafficking into the skin (42, 43) (e.g., CCR4 and CD103), (ii) homing into lymphoid organs (44, 45) (e.g., PSGL-1 and CD62L), (iii) homing to the intestinal mucosa (46, 47) (e.g., CCR9 and α4β7 integrin), and (iv) sites of inflammation (45, 48, 49) (e.g., PSGL-1, CD103, and CXCR3), the Treg could be effective in clinical studies aiming to treat various autoimmune-based and inflammatory disorders such as skin diseases, rheumatic diseases, intestinal inflammation, and graft-versus-host disease.

## Ethics Statement

This study was carried out in accordance with the recommendations of the local Review Board (IRB) of the Friedrich-Alexander Universität Erlangen-Nürnberg under IRB number 151_12B with written informed consent from all subjects. All subjects gave written informed consent in accordance with the Declaration of Helsinki. The protocol was approved by the local IRB of the Friedrich-Alexander Universität Erlangen-Nürnberg.

## Author Contributions

MW, DS, SR, CL, AF, IA, and CV performed experiments and analyzed data. GS and MN initiated the project. GS and BS-T acted as advisors during the GMP implementation. CV drafted the manuscript. MW, DS, SR, CL, AF, RA, CN, IA, AS, BS-T, MN, and GS critically revised the manuscript for intellectual content.

## Conflict of Interest Statement

The authors declare that the research was conducted in the absence of any commercial or financial relationships that could be construed as a potential conflict of interest.
