# Supplementary material for: Good Manufacturing Practice-Compliant Production and Lot-Release of Ex Vivo Expanded Regulatory T Cells As Basis for Treatment of Patients with Autoimmune and Inflammatory Disorders
Source: Front Immunol. 2017 Oct 26;8:1371. doi: 10.3389/fimmu.2017.01371 (PMC5662555; doi:10.3389/fimmu.2017.01371)

**Supplemental file 1**

**(A)** Exact absolute cell number per consistency run during expansion

|  | day0 | day7 | day11 | day14 | day14^b^ | day18 | day18^b^ | day21 |
| --- | --- | --- | --- | --- | --- | --- | --- | --- |
| CON1 | 60^a^ | 199 | 849 | 1020 | 434 | 2074 | 1000 | 1440 |
| CON2 | 30 | 113 | 573 | 297 | 50 | 159 | 159 | 528 |
| CON3 | 30 | 104 | 423 | 978 | 777 | 2512 | 1256 | 1392 |
| CON4 | 30 | 34 | 158 | 234 | 124 | 455 | 455 | 944 |

^a^Absolute cell number x 10^6^.

^b^To enable in-process evaluation, part of the expanded cells were removed at day 14 and day 18 from the cultures after cell harvest and count. Indicated cell numbers represent the amount of cells which remained in culture.

**(B)** Exact relative expansion rate per consistency run per measured time-point

|  | day7/day0 | day11/day7 | day14/day11 | day18/day14 | day21/day18 |
| --- | --- | --- | --- | --- | --- |
| CON1 | 3,3 | 4,3 | 1,2 | 4,8 | 1,4 |
| CON2 | 3,8 | 5,1 | 0,5 | 3,2 | 3,3 |
| CON3 | 3,5 | 4,1 | 2,3 | 3,2 | 1,1 |
| CON4 | 1,1 | 4,6 | 1,5 | 3,7 | 2,1 |

**(C)** Exact absolute expansion rate per consistency run per measured time-point

|  | day0 | day7 | day11 | day14 | day18 | day21 |
| --- | --- | --- | --- | --- | --- | --- |
| CON1 | 1,0 | 3,3 | 14,2 | 17,0 | 81,2 | **117,0** |
| CON2 | 1,0 | 3,8 | 19,1 | 9,9 | 31,5 | **104,5** |
| CON3 | 1,0 | 3,5 | 14,1 | 32,6 | 105,4 | **116,8** |
| CON4 | 1,0 | 1,1 | 5,3 | 7,8 | 28,6 | **59,4** |

(D) Exact absolute expansion rate per consistency run per measured time-point


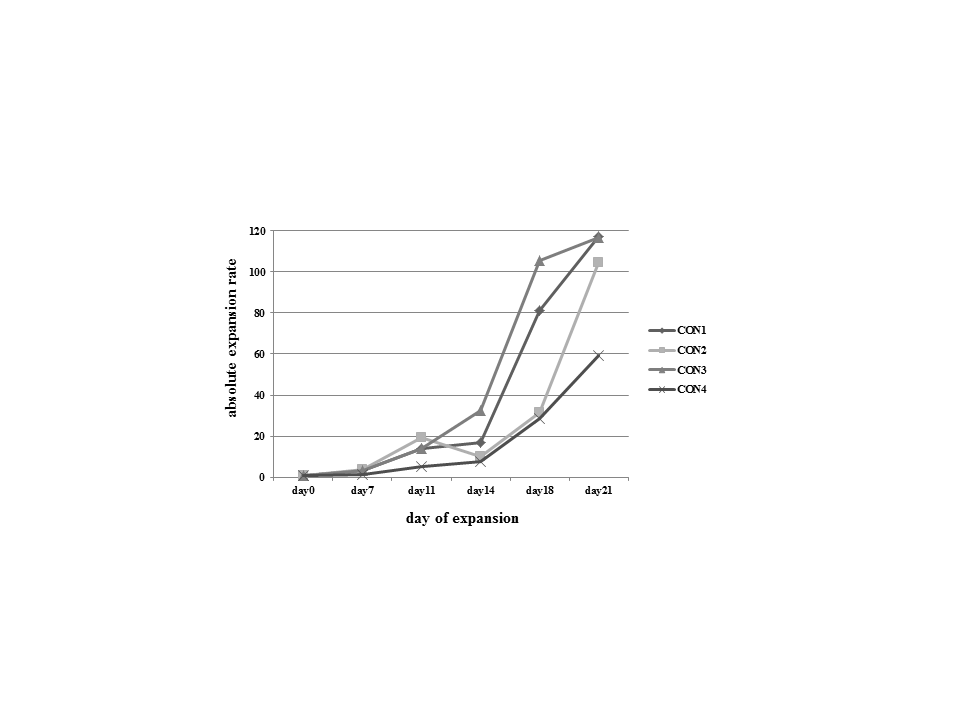

Supplement: Supplementary file 1 [file table_1.docx]
