# Supplementary material for: Good Manufacturing Practice-Compliant Production and Lot-Release of Ex Vivo Expanded Regulatory T Cells As Basis for Treatment of Patients with Autoimmune and Inflammatory Disorders
Source: Front Immunol. 2017 Oct 26;8:1371. doi: 10.3389/fimmu.2017.01371 (PMC5662555; doi:10.3389/fimmu.2017.01371)
Supplement: Supplementary file 2 [file table_2.docx]

**Supplemental file 2**

**
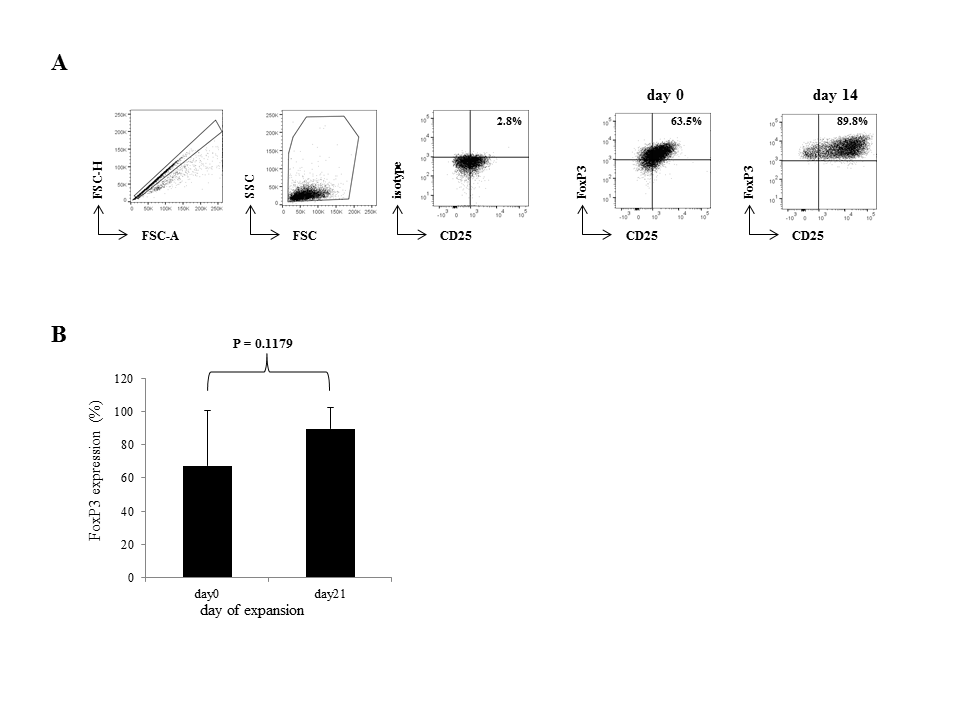
**

**Enriched CD25^+^ day 0 cells and day 21 expanded cells express intracellular FoxP3.** (A) Representative FACS plots gated on CD4^+^CD25^+^ cells showing FoxP3 expressing at day 0 after CD25^+^ cell enrichment and at expansion day 21. (B) Proportion of CD25^+^ cells expressing FoxP3 at day 0 after CD25^+^ cell enrichment and at expansion day 21 (n = 4).
