# Supplementary material for: Good Manufacturing Practice-Compliant Production and Lot-Release of Ex Vivo Expanded Regulatory T Cells As Basis for Treatment of Patients with Autoimmune and Inflammatory Disorders
Source: Front Immunol. 2017 Oct 26;8:1371. doi: 10.3389/fimmu.2017.01371 (PMC5662555; doi:10.3389/fimmu.2017.01371)
Supplement: Supplementary file 3 [file table_3.docx]

**Supplemental file 3**

**(A+B) Mean fluorescence intensity (MFI) of proliferated CD8^+^ cells based on all cell generations**


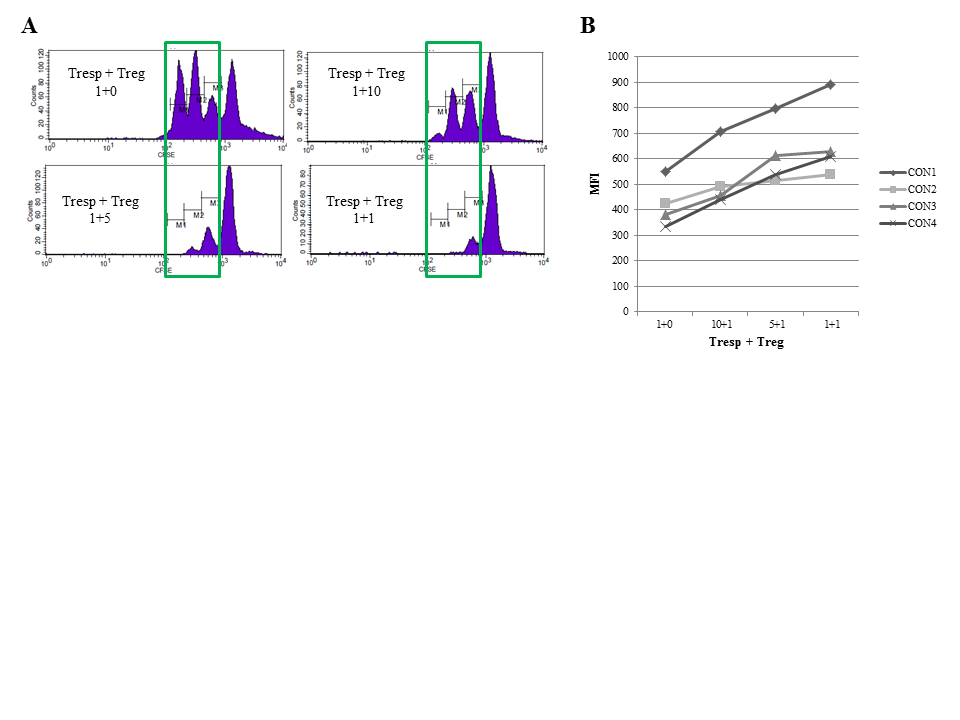


**(C) Absolute mean fluorescence intensity (MFI) values of proliferated CD8+ cells based on all cell generations (mean of triplicates)**

|  | Tresp+Treg | Tresp+Treg | Tresp+Treg | Tresp+Treg |
| --- | --- | --- | --- | --- |
|  | 1+0 | 10+1 | 5+1 | 1+1 |
| CON1 | 549 | 705 | 796 | 893 |
| CON2 | 423 | 491 | 516 | 539 |
| CON3 | 380 | 455 | 613 | 628 |
| CON4 | 334 | 440 | 539 | 610 |

**(D) Number of CD8^+^ cell generations**

|  | Tresp+Treg | Tresp+Treg | Tresp+Treg | Tresp+Treg |
| --- | --- | --- | --- | --- |
|  | 1+0 | 10+1 | 5+1 | 1+1 |
| CON1 | **3** | **2** | **2** | **1** |
| CON2 | **3** | **2** | **2** | **2** |
| CON3 | **3** | **3** | **2** | **2** |
| CON4 | **3** | **2** | **1** | **1** |

**Increased mean fluorescence intensity (MFI) values and a reduced absolute number of cell generations are associated with CD8^+^ cell suppression in the first generation of proliferated cells.** (A) Representative histograms gated on CD8^+^/CFSE^+^ responder cells showing MFI of responder cells in the absence of Treg (1+0) and in the presence of Treg at a ratio of 1+10, 1+5 and 1+1, as indicated. (B) Gradual increases in MFI values per consistency run at indicated ratios. (C) Absolute MFI values based on triplicate values per consistency run at indicated ratios. (D) Number of CD8+ cell generations per consistency run at indicated ratios.
